# Mutational Landscape of *CEBPA* in Mexican Pediatric Acute Myeloid Leukemia Patients: Prognostic Implications

**DOI:** 10.3389/fped.2022.899742

**Published:** 2022-07-11

**Authors:** Carolina Molina Garay, Karol Carrillo Sánchez, Luis Leonardo Flores Lagunes, Marco Jiménez Olivares, Anallely Muñoz Rivas, Beatríz Eugenia Villegas Torres, Hilario Flores Aguilar, Juan Carlos Núñez Enríquez, Elva Jiménez Hernández, Vilma Carolina Bekker Méndez, José Refugio Torres Nava, Janet Flores Lujano, Jorge Alfonso Martín Trejo, Minerva Mata Rocha, Aurora Medina Sansón, Laura Eugenia Espinoza Hernández, José Gabriel Peñaloza Gonzalez, Rosa Martha Espinosa Elizondo, Luz Victoria Flores Villegas, Raquel Amador Sanchez, María Luisa Pérez Saldívar, Omar Alejandro Sepúlveda Robles, Haydeé Rosas Vargas, Silvia Jiménez Morales, Patricia Galindo Delgado, Juan Manuel Mejía Aranguré, Carmen Alaez Verson

**Affiliations:** ^1^Laboratorio de Diagnóstico Genómico, Instituto Nacional de Medicina Genómica (INMEGEN), Mexico City, Mexico; ^2^Instituto de Diagnóstico y Referencia Epidemiológicos (InDRE), Mexico City, Mexico; ^3^Unidad de Investigación Médica en Epidemiología Clínica, UMAE Hospital de Pediatría, Centro Médico Nacional “Siglo XXI”, Instituto Mexicano del Seguro Social (IMSS), Mexico City, Mexico; ^4^Servicio de Hematología Pediátrica, Hospital General “Gaudencio González Garza”, Centro Médico Nacional (CMN) “La Raza”, Instituto Mexicano del Seguro Social (IMSS), Mexico City, Mexico; ^5^Unidad de Investigación Médica en Inmunología e Infectología, Hospital de Infectología “Dr. Daniel Méndez Hernández”, “La Raza”, Instituto Mexicano del Seguro Social (IMSS), Mexico City, Mexico; ^6^Servicio de Oncología, Hospital Pediátrico de Moctezuma, Secretaria de Salud del D.F., Mexico City, Mexico; ^7^Servicio de Hematología Pediátrica, UMAE Hospital de Pediatría, Centro Médico Nacional (CMN) “Siglo XXI”, Instituto Mexicano del Seguro Social (IMSS), Mexico City, Mexico; ^8^Servicio de Hemato-Oncología, Hospital Infantil de México Federico Gómez, Secretaria de Salud (SSa), Mexico City, Mexico; ^9^Servicio de Onco-Pediatría, Hospital Juárez de México, Secretaria de Salud (SSa), Mexico City, Mexico; ^10^Servicio de Hematología Pediátrica, Hospital General de México, Secretaria de Salud (SSa), Mexico City, Mexico; ^11^Servicio de Hematología Pediátrica, Centro Médico Nacional (CMN) “20 de Noviembre”, Instituto de Seguridad Social al Servicio de los Trabajadores del Estado (ISSSTE), Mexico City, Mexico; ^12^Hospital General Regional No. 1 “Carlos McGregor Sánchez Navarro”, Instituto Mexicano del Seguro Social (IMSS), Mexico City, Mexico; ^13^Unidad de Investigación Médica en Genética Humana, UMAE Hospital de Pediatría, Centro Médico Nacional (CMN) “Siglo XXI”, Instituto Mexicano del Seguro Social (IMSS), Mexico City, Mexico; ^14^Laboratorio de Genómica del Cáncer, Instituto Nacional de Medicina Genómica (Inmegen), Mexico City, Mexico; ^15^Hospital Central Sur de Alta Especialidad de Pemex, Mexico City, Mexico; ^16^Facultad de Medicina, Universidad Nacional Autónoma de México, Mexico City, Mexico

**Keywords:** *CEBPA*, pediatric, Mexican, AML, survival, risk-stratification

## Abstract

**Background:**

In Mexico, the incidence of acute myeloid leukemia (AML) has increased in the last few years. Mortality is higher than in developed countries, even though the same chemotherapy protocols are used. CCAAT Enhancer Binding Protein Alpha (*CEBPA*) mutations are recurrent in AML, influence prognosis, and help to define treatment strategies. *CEBPA* mutational profiles and their clinical implications have not been evaluated in Mexican pediatric AML patients.

**Aim of the Study:**

To identify the mutational landscape of the *CEBPA* gene in pediatric patients with *de novo* AML and assess its influence on clinical features and overall survival (OS).

**Materials and Methods:**

DNA was extracted from bone marrow aspirates at diagnosis. Targeted massive parallel sequencing of *CEBPA* was performed in 80 patients.

**Results:**

*CEBPA* was mutated in 12.5% (10/80) of patients. Frameshifts at the N-terminal region were the most common mutations 57.14% (8/14). *CEBPA* biallelic (*CEBPA*^BI^) mutations were identified in five patients. M2 subtype was the most common in *CEBPA* positive patients (*CEBPA*^POS^) (*p* = 0.009); 50% of the *CEBPA*^POS^ patients had a WBC count > 100,000 at diagnosis (*p* = 0.004). OS > 1 year was significantly better in *CEBPA* negative (*CEBPA*^NEG^) patients (*p* = 0.0001). *CEBPA*^POS^ patients (either bi- or monoallelic) had a significantly lower OS (*p* = 0.002). Concurrent mutations in *FLT3*, *CSF3R*, and *WT1* genes were found in *CEBPA*^POS^ individuals. Their contribution to poor OS cannot be ruled out.

**Conclusion:**

CEBPA mutational profiles in Mexican pediatric AML patients and their clinical implications were evaluated for the first time. The frequency of *CEBPA*^POS^ was in the range reported for pediatric AML (4.5–15%). *CEBPA* mutations showed a negative impact on OS as opposed to the results of other studies.

## Introduction

Acute myeloid leukemia (AML) is characterized by uncontrolled proliferation and accumulation of immature myeloid precursor cells in the bone marrow, which leads to impaired hematopoiesis and bone marrow failure (1).

Acute myeloid leukemia is the second most common cancer in Mexican children. Its incidence has increased in the last years, and its mortality is higher than in developed countries even though the same chemotherapy protocols are used (2–4). AML accounts for 15–20% of leukemia-related mortality (5). Only 30% of patients achieve complete remission. This figure is significantly lower than the 90–95% reported literature (6). Mortality at the beginning of treatment is also higher than expected (7).

Several extensive sequencing studies on AML revealed the genetic heterogeneity of the disease: on average, 13 mutations were detected per patient, and at least 23 recurrently mutated genes were found (8). Some recurrent chromosomal translocations and somatic gene mutations are already included in clinical guidelines as biomarkers to improve disease classification, prognostic categorization, and definition of treatment strategies (9, 10).

CCAAT Enhancer Binding Protein Alpha (*CEBPA*) is one of the recurrently mutated genes in both adult (7–16%) (11, 12) and pediatric (5–15%) AML patients (13–15). The 2016 revision to the World Health Organization classification of myeloid neoplasms includes two distinct *CEBPA*-related disease entities: AML with biallelic *CEBPA* mutations (*CEBPA*^BI^) and AML with germline *CEBPA* mutations. Germline *CEBPA* mutations at N- and C-terminal protein domains have been described. They may lead to familial *CEBPA*^BI^ AML after acquiring a second hit in the *CEBPA* gene (9).

CEBPA encodes the CCAAT/enhancer-binding protein alpha, a lineage-specific basic leucine zipper (bZIP) transcription factor required to form myeloid progenitors from multipotent hematopoietic stem cells. It is expressed at high levels during myeloid cell differentiation. It binds to the promoters of multiple genes during myeloid linage maturation (16).

The *CEBPA* protein contains two transactivation domains (TADs) at its N-terminus, a DNA binding domain, and a basic leucine zipper at the C-terminus responsible for DNA binding and dimerization. *CEBPA* gene is located on chromosome 19q13.1 and has only one exon. It is transcribed into a single mRNA, translated into two isoforms by alternative start codon usage: a 42-kDa full isoform (p42) or a truncated 30-kDa (p30) isoform lacking the TAD1 domain. Both isoforms can make homo- or heterodimers with other proteins and participate in myeloid differentiation and other cellular processes (12).

As in most Latin American countries, extensive tumoral profiling is not routinely performed in Mexico; therefore, the information about the mutational profiles and their possible impact on outcomes in pediatric or adult patients is scarce or not available. Using real-time PCR methodology or FISH, molecular testing is limited to the most common fusions described in AML and acute lymphocytic leukemia. This study aims to explore the mutational profile of the *CEBPA* gene in a group of pediatric *de novo* AML patients and to evaluate the possible impact on clinical features and overall survival (OS).

## Materials and Methods

### Population

This study analyzed 80 patients with *de novo* AML. They were diagnosed between March 2010 and March 2018. The diagnosis was made at each institution by bone marrow aspirate and immunophenotype. Bone marrow samples were obtained at the time of diagnosis and submitted to the Mexican Inter-Institutional Group for Identifying Childhood Leukemia Causes in Mexico City. Data were collected from medical charts, including sex, age, peripheral white blood cell count, percentage of bone marrow blasts at diagnosis, FAB (French-American-British) classification, and treatment protocol. The clinical features of the analyzed patients had been previously reported in Molina et al. (17).

Risk classification at diagnosis was assigned based mainly on morphology; in most public hospitals in Mexico City, cytogenetics and minimal residual disease detection are unavailable. The risk groups were established as follows: standard-risk group: M1, M2, and M4 (with at least 3% of eosinophils); high-risk group: M4 (with less than 3% of eosinophils) and M5. Additionally, in one hospital, more than 5% of blasts in bone marrow on day 15 was used to identify patient with high-risk features. In M3 patients low-risk group includes patients with white blood cell count (WBC) < 10 × 10^9^ and platelets > 40 × 10^9^; for intermedia-risk group WBC count < 10 × 10^9^ and platelets < 40 × 10^9^ and for high-risk group WBC ≥ 10 × 10^9^.

Patients were classified with an intermediate risk when MRD level by flow cytometry was >0.1% after course 1, but fell to <0.1% after course 2. Some child’s parents covered this test in a private laboratory.

The ethics and scientific review boards of the National Institute of Genomic Medicine, Mexico City, Mexico, approved this study (document number 28-2015-1). All human samples and clinical information were approved for the present study. The children’s parents signed the informed consent obtained according to the Declaration of Helsinki.

### DNA Extraction

The DNA was extracted from bone marrow samples with Maxwell^®^ 16 Blood DNA Purification Kit (Promega, Madison, WI, United States) according to the manufacturer’s recommendations. The DNA purity and concentration were measured with NanoDrop 1000 spectrophotometer (Thermo Fisher Scientific, Waltham, MA, United States) and Qubit fluorometer (Thermo Fisher Scientific, Waltham, MA, United States).

### Next-Generation Sequencing

CEBPA sequencing was performed with the “Myeloid solution” panel by Sophia Genetics (Sophia Genetics SA, Saint-Sulpice, Switzerland). Library preparation and sequencing were performed according to the manufacturer’s protocol. Libraries were pooled and sequenced on a MiSeq System v3 chemistry (Illumina, San Diego, CA, United States). Sequence data were analyzed with the Sophia DDM^®^ software version 5.2.7.1 (Sophia Genetics SA, Saint-Sulpice, Switzerland). Deep sequencing was greater than 500X for all the target regions. Variant Fraction (VF) was calculated for each mutation by dividing the number of sequencing reads showing the mutation by the total sequencing read at the mutation position.

### Statistical Analysis

The statistical analysis was performed as described previously (17). For dichotomic variables, chi-square or two-sided’ Fisher’s exact test was used to compare proportions among different groups. A non-parametric Mann-Whitney *U* test was applied for continuous variables, a *p*-value < 0.05 was considered statistically significant. All calculations were performed with the SPSS software package, SPSS v21 (Chicago, IL, United States).

The Kaplan-Meier method (18) was used to assess overall survival (OS). The log-rank test was used to evaluate differences between survival distributions with a 95% confidence interval (CI). The OS time was calculated from the day diagnosis was confirmed to either the last follow-up or death from any cause. Patients who did not experience an event were censored at the last follow-up. Those who did not attend follow-up appointments were censored at the date of the last known contact.

The maternal years of education were used as socioeconomic status (SES) indicator to evaluate if it impacted the inferior OS observed. The patients were further categorized in *CEBPA* (positive/negative) and death (yes/no) to identify possible associations. As per the categorization used by the Childhood Leukemia International Consortium, SES was assigned as follows: [0–9 years (low SES), 9.1–12.9 years (reference category), ≥13 years of education (high SES)] (19, 20). The exact Fisher’s test was used to assess the difference between groups.

## Results

### Demographic and Biological Characteristics of the Patients

Demographic, clinical, and the main biological characteristics of the patients were previously reported (17). Fifty-five percent of the patients were male. The age at diagnosis was similar in both sexes; the mean age at diagnosis was 9.3 years (range 0.4–17.5). Acute promyelocytic leukemia (APL) M3 was the most prevalent subtype (36.3%), followed by M2 (33.8%). M0, M5, and M6 were the less common subtypes. The mean OS in the study population was 1.95 years.

The patients were treated based on one of the following four protocols: BFM-1998, BFM-2001 (Berlin-Frankfurt-Münster 1998 and 2001), NOPHO-AML93 (Nordic Society of Pediatric Hematology and Oncology), or PETHEMA-APL-05 (Spanish Program of Treatments in Hematology). APL patients were treated according to the PETHEMA-APL-05 protocol. None of the patients received an allogeneic or autologous bone marrow transplant. Treatment information and the impact of treatment on OS have been previously reported (17).

### *CCAAT Enhancer Binding Protein Alpha* Mutational Profile in Mexican Pediatric Acute Myeloid Leukemia Patients

**FIGURE 1:**
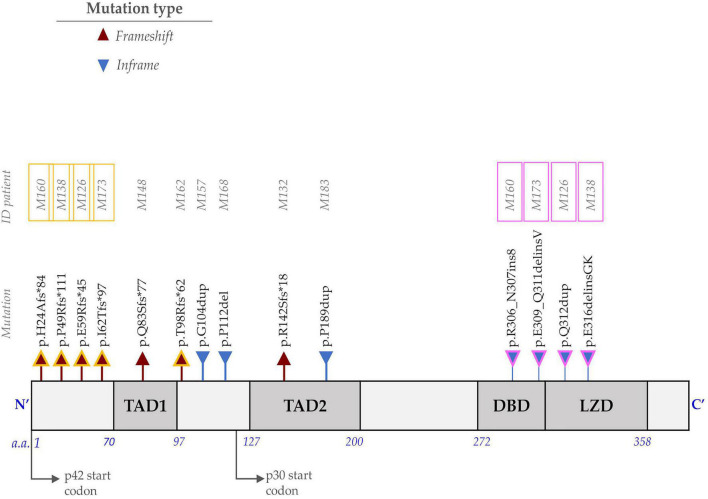
Representation of the protein encoded by the CCAAT Enhancer Binding Protein Alpha (*CEBPA*) gene and the identified mutations. The N-terminus consists of two transactivation domains (TAD1 and TAD2). The carboxyl end (C-terminus) includes the DBD (DNA-binding domain) and the LZD (leucine zipper domain). The frameshift mutations (red triangle) at the N-terminus affect the translation of the p42 isoform and favor the overexpression of the p30 isoform. In-frame mutations (blue triangle) at the C-terminus alter the DNA binding or dimerization process. Individuals with *CEBPA*^BI^ (M160, M138, M126, and M173) have one mutation at the N-terminus (outlined in yellow) and one at the C-terminus (outlined in pink), whereas M162 is homozygous for p.Thr98Argfs*62.

*CEBPA* was mutated in 12.5% (10/80) of the cases (*CEBPA*^POS^). A total of 14 different mutations were identified. The mutational profile is shown in Table 1 and Figure 1. The most common mutations were frameshifts located at the N-terminus of the protein 57.14% (8/14). Biallelic *CEBPA* mutations (*CEBPA*^BI^) were identified in five patients, which accounted for 50% of the *CEBPA*^POS^ cases. All identified mutations were uploaded to the ClinVar database (accession numbers for submission SUB11180409 are SCV002104196-SCV002104210)^1^.

**TABLE 1:** Mutational overview of the CEBPA gene in Mexican patients with pediatric AML.

Patient ID	Coding consequence	cDNA	Protein consequence	VF (%)	COSMIC ID	dbSNP	Co-occurring mutations
M160	frameshift	c.68dupC	p.His24Alafs*84	39.2	COSM18922	rs137852729	*NRAS*, *ZRSR2*, *EZH2*
	inframe_24	c.918_919ins24	p.Arg306_Asn307ins8	42.8	Novel	/	
M138	frameshift	c.146delC	p.Pro49Argfs*111	50.5	COSM5064965	/	*WT1*, *CALR*, *PTPN11*
	inframe_3	c.946_947insGGA	p.Glu316delinsGlyLys	48.2	Novel	/	
M173	frameshift	c.180_183delGTCC	p.Ile62Thrfs*97	44.8	Novel	/	*ASXL1*
	inframe_6	c.926_932delAGACGCAinsT	p.Glu309_Gln311delinsVal	42.7	Novel	/	
M126	frameshift	c.174_184delCGAGACGTCCA	p.Glu59Argfs*45	49.2	COSM29261	/	*FLT3*, *CSF3R*, *CALR*, *PTPN11*
	inframe_3	c.934_936dupCAG	p.Gln312dup	49.6	COSM18466	/	
M162	frameshift	c.292delA	p.Thr98Argfs*62	94.4	Novel	/	*CSF3R*
M148	frameshift	c.247delC	p.Gln83Serfs*77	42.5	COSM1375	/	*FLT3*, *WT1*, *PTPN11*
M132	frameshift	c.426delG	p.Arg142Serfs*18	48.7	Novel	/	*IDH2*
M157	inframe_3	c.311_313dupGCG	p.Gly104dup	1.8	/	rs780345232	*RUNX1*, *PTPN11*
M168	inframe_3	c.334_336delCCC	p.Pro112del	1.3	Novel	/	*ETV6*
M183	inframe_3	c.564_566dupGCC	p.Pro189dup	1.2	Novel	/	*FLT3*, *TET2*, *RUNX1*, *CBL*

*VF, variant fraction; COSMIC, catalogue of somatic mutations in cancer; NM_004364 was used for variant annotation.*

Five germline variants were also identified: His191His (27.5%), Thr230Thr (5%), Hist195_Pro196 (5%), and Pro204Pro (1.2%). They were classified as benign or likely benign according to the ACMG criteria (21). The variant pGly223Ser located between the TAD2 and BDB domains was identified in one individual (1.2%, VF = 47.5%). It is listed in the ClinVar database (Variation ID 239926) and shows conflict regarding its clinical significance (uncertain significance vs. likely benign). According to the gnomAD v3 population database, its global frequency is 0.0004189 (58 of 138,460 alleles), with a maximum allele frequency of 0.004221 (56 of 13,266) in the Latino subpopulation. It has been identified in adults only^2^ (as of March 2022). The frequency in a local exome database of Mexican individuals is 1.1%. This frequency exceeds the prevalence of a pathogenic variant causing *CEBPA*-associated familial AML (22). The variant has not been reported in cases of familial AML. It is predicted benign for most *in silico* algorithms, although no functional studies have been performed. Considering the available information, we classified this variant as likely benign.

### Distribution of Demographic and Biological Features Between *CEBPA*^POS^ and *CEBPA*^NEG^ Patients

Sex distribution, FAB classification, treatment protocol, risk assessment, WBC count, age, and blast percentage in bone marrow at diagnosis were stratified according to the presence of *CEBPA* mutations. The results are shown in Table 2. A non-significant (NS) higher proportion of male patients was found in the *CEBPA*^POS^ subgroup (60 vs. 54.3% in *CEBPA*^NEG^ group, *p* = NS). Statistically significant differences were found in the FAB subtype distribution. M2 was the most common among *CEBPA*^POS^ patients (70 vs. 28.6% in *CEBPA*^NEG^ *p* = 0.009). *CEBPA*^POS^ patients had significantly higher WBC counts at diagnosis: 50% of *CEBPA*^POS^ patients had a WBC count > 100,000, compared with 12.9% in the *CEBPA*^NEG^ group (*p* = 0.004).

**TABLE 2:** Distribution of demographic and biological features between *CEBPA*^POS^ and *CEBPA*^NEG^ patients.

	*CEBPA*^POS^ N = 10		*CEBPA*^NEG^ N = 70		*p*
Sex	^a^N	%	N	%	
Female	4	40	32	45.7	
Male	6	60	38	54.3	
**Mean age at ^b^Dx**	**Years**	**Range**	**Years**	**Range**	
Female	9.24	4.9 - 10.1	9.4	0.4 - 17.5	
Male	9.7	5.3 - 15.9	9.1	1.3 - 16.6	
Total	9.6	4.9 - 15.9	9.3	0.4 - 17.5	
**Mean ^c^BM blast at Dx**	**Mean (%)**	**Range**	**Mean (%)**	**Range**	
	84.3	65 - 98	75.9	23 - 100	
**^d^WBC count at Dx/mm3**	**N**	**%**	**N**	**%**	
<11,000	2	20	29	41.4	
11,000–100,000	3	30	32	45.7	
>100,000	5	50	9	12.9	*0*.*004*
**^d^WBC median at Dx/mm3**	**N**	**Median mm3**	**N**	**Median mm3**	
<11,000	2	5000	29	4300	
11,000–100,000	3	42400	32	34450	
>100,000	5	249000	9	146100	
Total	10	117630	70	18830	
**Mean overall survival**	**N**	**Years**	**N**	**Years**	
OS	10	0.9	69	1.97	
OS ≤ 1 year	5 (50%)	0.3	25 (36.2%)	0.35	
OS > 1 year	5 (50%)	1.8	44 (63.8%)	3.1	*0*.*0001*
OS ≤ 2 year	8 (80%)	0.8	40 (58%)	0.8	
OS > 2 year	2 (20%)	2.1	29 (42%)	3.9	*0*.*0258*
**^e^FAB subtypes**	**N**	**%**	**N**	**%**	
M0	0	0	1	1.4	
M1	1	10	8	11.4	
M2	7	70	20	28.6	*0*.*009*
M3	2	20	27	38.6	
M4	0	0	12	17.1	
M5	0	0	1	1.4	
M6	0	0	1	1.4	
**Treatment protocols**	**N**	**%**	**N**	**%**	
^f^BFM-1998	4	40	21	30	
BFM-2001	2	20	4	5.7	
^g^NOPHO-AML93	2	20	18	25.7	
^h^PETHEMA-APL-05	2	20	27	38.6	
**Risk classification at Dx.**	**N**	**%**	**N**	**%**	
Standard	0	0	6	8.6	
Intermediate	0	0	4	5.7	
High	5	50	39	55.7	
Not classified	5	50	21	30	
**Achievement of ^i^CR**	**N**	**%**	**N**	**%**	
	10	100	67	95.7	
**Relapsed**	**N**	**%**	**N**	**%**	
	3	30	4	5.7%	

*^a^N, number of patients; ^b^Dx, diagnosis; ^c^BM, bone marrow; ^d^WBC, white blood cell; ^e^FAB, French-American-British classification; ^f^BFM, Berlin-Frankfurt-Münster; ^g^NOPHO, Nordic Society of Pediatric Hematology and Oncology; ^h^PETHEMA, Spanish Program of Treatments in Hematology; ^i^CR, complete remission assessed on day 28. P value < 0.05 was considered statistically significant.*

### Impact of *CEBPA* Mutation on Overall Survival

A significantly higher mean OS was observed in *CEBPA*^NEG^ patients considering 1 year after diagnosis (3.1 years in *CEBPA*^NEG^ vs. 1.8 years in *CEBPA*^POS^, *p* = 0.0001) or 2 years (2.1 years in *CEBPA*^POS^ vs. 3.9 years in *CEBPA*^NEG^, *p* = 0.0258).

**FIGURE 2:**
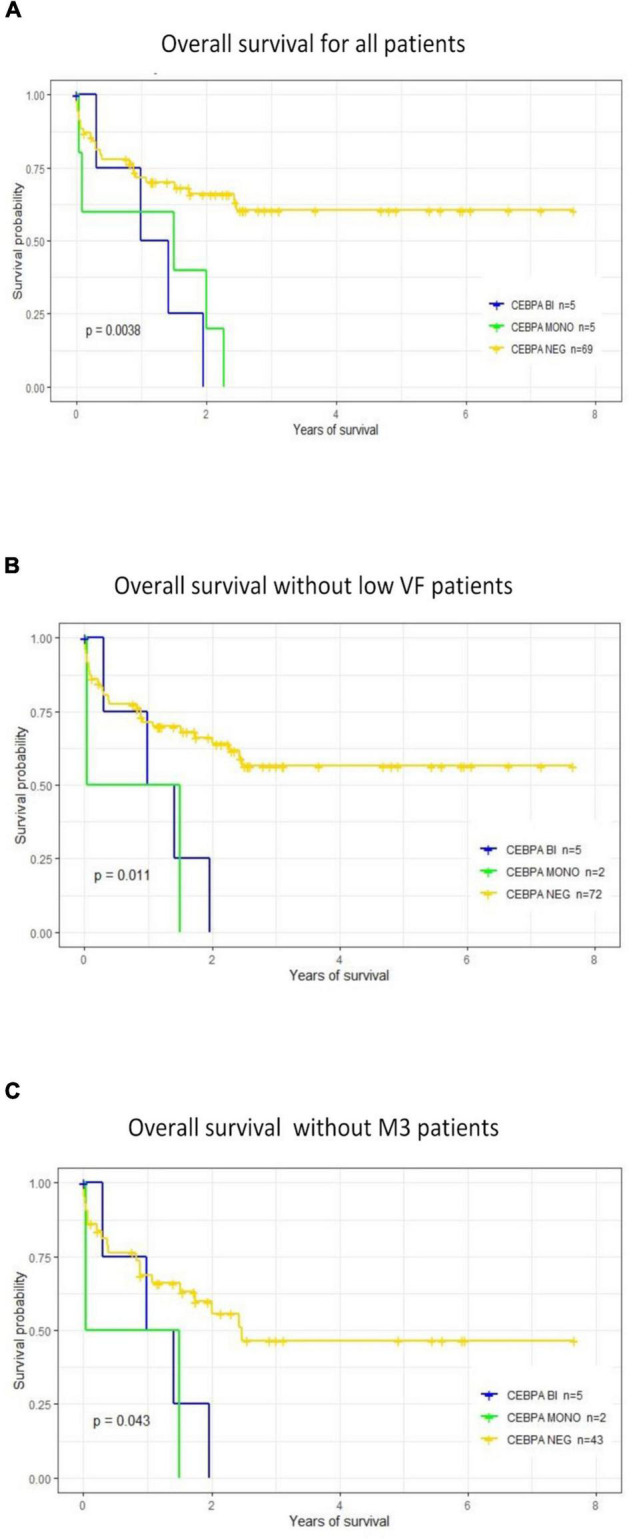
Prognostic impact of CCAAT Enhancer Binding Protein Alpha (*CEBPA*) mutations on overall survival. **(A)** OS was analyzed considering the following groups: *CEBPA*^MONO^ or *CEBPA*^BI^ vs. *CEBPA*^NEG^. Groups with mono or biallelic mutations showed a significantly lower OS than patients without *CEBPA* mutations (*CEBPA*^NEG^). **(B)** OS was analyzed after removing the three patients with *CEBPA*^MONO^ and VF < 2%. Results are similar; OS is significantly lower in the *CEBPA* mutated subgroup. **(C)** Comparing OS between patients positive for *CEBPA* (*CEBPA*^BI^ or *CEBPA*^MONO^) vs. *CEBPA*^NEG^, after removing patients classified as AML-M3. There is a significantly lower OS in the *CEBPA*^POS^ subgroups.

The impact of *CEBPA* mutation on OS was analyzed by comparing three subgroups of patients according to *CEBPA* status: *CEBPA*^MONO^ (individuals with monoallelic mutations), *CEBPA*^BI^, and *CEBPA*^NEG^. The results are shown in Figures 2A–C. The OS significantly decreased in patients with *CEBPA* mutations compared with the CEBPA^NEG^ group (*p* = 0.002). No differences were found in OS between patients with *CEBPA*^MONO^ and *CEBPA*^BI^. Similar results were observed after removing from the analysis the three *CEBPA*^MONO^ patients with CEBPA mutation VF < 2% (patients M157, M168, and M183). A significant decrease in OS was observed (*p* = 0.009), as shown in Figure 2B. OS analysis was performed after removing the M3 patients from the *CEBPA*^NEG^ mutated group. The result still showed a significantly lower OS in the *CEBPA*^POS^ group (*p* = 0.04; Figure 2C).

No statistically significant association was detected between *CEBPA*^POS^ cases and a low SES (*p* = 0.99) or between a low SES and a poor outcome (death) (*p* = 0.76; Results of the analysis are shown in Supplementary Table 1).

## Discussion

This study is the first to evaluate the mutational landscape of the *CEBPA* gene and its clinical impact on Mexican patients with *de novo* AML. The proportion of *CEBPA*^POS^ patients was 12.5%, close to the 14.9% described in 315 AML pediatric patients (15) and the range of frequency reported in the adult population (7–16%) (15, 23). Of all identified mutations, 57.14% (8/14) were located at the N-terminus. Most of them were frameshifts predicted to result in the premature stop of the wild-type p42 translation while preserving the translation of a short p30 isoform by using the second ATG present downstream and overexpressing the p30 isoform (24). Mutation p.R142Sfs*18 is located downstream of the p30 start codon at the TAD2 domain and would suppress the expression of p42 and p30 isoform from this *CEBPA* mutated allele. Mutations in the C-terminus portion were less common, 28.57% (4/14), and were insertion/deletions that did not affect the gene reading frame. These mutations are distributed in both DNA binding and leucine zipper domains affecting the dimerization or the binding to the DNA processes (24).

A high proportion of the identified mutations are new; only 7 out of 14 had been previously reported in the COSMIC^3^ or dbSNP databases^4^ (as of March 2022). All the identified variants were uploaded to the ClinVar database. The high proportion of novel variants identified in this small subgroup of *CEBPA*^POS^ patients supports the importance of research focused on ethnically diverse populations of non-Caucasian ethnicities. A more comprehensive data on mutational profiles on cancer driver genes would contribute to a better understanding of the disparities in cancer outcomes observed by race/ethnicity (25).

All the novel mutations with VF > 40% were classified as pathogenic or likely pathogenic according to ACMG criteria (Supplementary Table 2). No functional *in vitro* analyses were performed to confirm their pathogenicity. However, most novel mutations are frameshift deletion (patients M173, M162, and M132) or inframe insertion (M160, M138, and M173). According to the COSMIC database statistics for *CEBPA* gene^5^, these types of mutations are the most frequent, representing 46% of all kinds of mutations in *CEBPA* across different tumors (Supplementary Figure 1). Considering this information and the published literature analyzing *CEBPA* mutations and their consequence, we thought it is improbable that these variants were not deleterious for the *CEBPA* protein functioning. The two variants with VF < 2% were classified as Variant of Uncertain Significance (VUS); these variants were removed from the survival analysis.

Half of the *CEBPA*^POS^ patients had *CEBPA*^MONO^, similar to the findings in Japanese children with AML (55.5%) (15). The leukemogenic mechanism for monoallelic *CEBPA* is not clearly established. It has been proposed that monoallelic mutations favor a preleukemic stem cell that, through a multistep clonal evolutionary process, acquires additional cooperating mutations and fully develops a leukemic clone (26).

Pabst et al. described that around 60% of AML patients had biallelic mutated *CEBPA* (27). This proportion was lower in our series; *CEBPA*^BI^ mutations were found in 50% (5/10) of the cases. One patient was homozygous for a frameshift mutation affecting the TAD1 domain (M162). The other four were compound heterozygous for one variant located at the N-terminus and the other at the C-terminus region, the most common locations for biallelic mutations. Compound heterozygous and homozygous mutations have been previously identified in biallelic mutated *CEBPA* AML patients (15).

Biallelic mutation induces a biallelic expression of aberrant p30 isoforms; the residual p30 activity inhibits the remaining p42 protein in a dominant-negative manner and affects the myeloid differentiation process (27). Some data obtained in animal models propose that truncated p30 isoforms may also act as a gain of functions allele favoring the leukemogenesis. Knockout p42 mice with preserved p30 expression develop AML with complete penetrance; however, when both isoform expressions were abolished, AML was not developed (28, 29).

Germline *CEBPA* mutations induced a leukemia predisposition syndrome called familial *CEBPA*-mutated AML. They have been identified in 4–15% of *CEBPA*^POS^ AML (11, 30, 31). Germline mutations could be located at both the N- or C-terminus (11, 14, 32, 33), having a VF between 40 and 60% in heterozygous individuals. Mutations with VF close to the expected heterozygous value need further evaluation. DNA extracted from no hematopoietic tissue could be used to clarify its origin (34). In this study, patients with biallelic mutations showed VF close to the expected value for germline variant. However, no skin biopsy or remission samples were available for further evaluation. Although no AML family history was identified in the clinical records of our *CEBPA*^BI^ patients, germline origin cannot be confidently excluded based only on family history due to incomplete AML penetrance, incomplete family history, or the possibility of a *de novo* germline variant.

### Impact of *CEBPA* Mutation on Clinical Features and Overall Survival

*CEBPA* mutations have been found more frequently among FAB M1 and M2 patients (35, 36). This was also true in our series (Table 2); M1 or M2 were present in 80% of the *CEBPA*^POS^ patients vs. 40% of the *CEBPA*^NEG^ subgroup (*p* = 0.01). However, a statistically significant difference was observed in the M2 distribution (*p* = 0.009). *CEBPA*^POS^ patients had a significantly higher WBC count at diagnosis; 50% of the *CEBPA*^POS^ patients had a WBC count > 100,000, compared with 12.9% in the *CEBPA*^NEG^ subgroup. Hyperleukocytosis at diagnosis has been associated with an increased risk for relapse in adults with *CEBPA*^BI^ AML (36). Due to the small number of patients in each subgroup, the statistical power is low to compare the distribution of clinical features between *CEBPA*^BI^ and *CEBPA*^MONO^.

*CEBPA* mutations were associated with a significantly lower OS but without significant differences between mono or biallelic *CEBPA* mutated patients. The mean OS in *CEBPA*^BI^ patients was 2 months longer than in *CEBPA*^MONO^ (11.2 vs. 9.27 months *p* = NS).

Considering that the biological impact of mutations with VF < 2%, classified as VUS, that may belong to a small subclone, would be different from those present in a higher proportion in the dominant clone, we evaluated the effect of *CEBPA* mutations in OS after removing the three patients with low allelic fractions. The result was similar to the one obtained taking into account the whole *CEBPA*^POS^ group (Figure 2B).

Several studies have assessed the clinical impact of *CEBPA* biallelic mutations on the survival of pediatric patients to determine if they are useful biomarkers of favorable-risk AML, as observed in adults. The findings of the most representative studies are summarized in Table 3. In contrast with our results, none of those studies have reported a negative effect of *CEBPA* mutations on OS; they found no impact of *CEBPA* mutations or a positive effect mainly for *CEBPA*^BI^ mutations.

**TABLE 3:** Results of different studies that evaluated the effect of CCAAT Enhancer Binding Protein Alpha (*CEBPA*) mutations in clinical outcomes of pediatric acute myeloid leukemia (AML) patients.

Year of publication	Total patients	*CEBPA* ^POS^	Clinical impact of *CEBPA* mutations	References
2009	847	4.5%	*CEBPA*^POS^ improved OS (at 5 years HR = 0.40, *p* = 0.023) and EFS (HR = 0.47, p = 0.01) No differences between *CEBPA*^BI^ and *CEBPA*^MONO^	Ho et al. (47)
2011	252	7.9%	*CEBPA*^BI^ had significantly better OS as compared with *CEBPA^MONO^* and *CEBPA*^NEG^ *CEBPA*^BI^ was an independent marker for OS in multivariate analysis	Hollink et al. (14)
2011	170	6.0%	No impact on OS or EFS of *CEBPA* mutations	Staffas et al. (51)
2014	315	14.9%	*CEBPA*^MONO^ and *CEBPA*^BI^ are independent favorable prognostic factors for EFS *CEBPA*^BI^ was an independent favorable prognostic factor for OS	Matsuo et al. (15)
2019	2958	5.4%	There were no differences in remission rates, OS, or EFS between *CEBPA*^MONO^ and *CEBPA*^BI^ patients	Tarlock et al. (52)

*OS, overall survival; EFS, event-free survival.*

Heterogeneity in relapse rates and survival outcomes has been reported in *CEBPA*^POS^ patients despite the favorable impact of *CEBPA*^BI^ mutations on OS (36). Concurrent mutations in other genes and a differential impact of *CEBPA* mutations according to their location would contribute to the observed heterogeneity (15, 37). We searched for concurrent mutations in *FLT3*, *NPM1*, *CSF3R* among *CEBPA*^POS^ patients; however, statistical tests could not be run due to the small sample size.

*FLT3* was mutated with VF > 30% in 3 out of 10 *CEBPA*^POS^ patients (one *CEBPA*^BI^ and two *CEBPA*^MONO^). We had previously reported that *FLT3* mutations have a negative impact on clinical outcomes in Mexican pediatric AML patients, and OS is significantly lower in patients with *FLT3* mutations than in *FLT3*^NEG^ (17, 37). Akin et al. reported that 2 out of 3 patients who carried concurrent *CEBPA* and *FLT3* mutations died during treatment (38). A negative effect of *FLT3* mutations on the *CEBPA*^BI^ positive effect has been observed in adult patients. However, a similar frequency of *FLT3-*ITD between mono and biallelic *CEBPA* mutated patients with no impact on OS has been found in children (14).

None of the *CEBPA*^POS^ patients harbored *NPM1* mutations. This supports the idea that *CEBPA* and *NPM1* mutations are mutually exclusive (39). The frequency of *NPM1* in our series (1.2%) was much lower than previously reported (6–8%) (40–43).

*CSF3R* was mutated in two *CEBPA*^POS^ individuals; p.Thr618Ile with VF = 48.6% was identified in one of them. This activating mutation occurs in the membrane-proximal region of the colony-stimulating factor 3 receptor. The mutation produces ligand-independent receptor activation, a hallmark of chronic neutrophilic leukemia (44). Concurrent *CSF3R* mutations in pediatric *CEBPA*^BI^ AML were associated with significantly poorer relapse-free survival than wild-type *CSF3R*; however, OS was not significantly different (45). A similar impact of *CSF3R* mutations on *CEBPA*^BI^ groups has been reported in adults (46). A pathogenic mutation in *WT1* with VF = 87% was also identified in one *CEBPA*^BI^ patient. Ho et al. also found three *WT1-*mutated patients in the *CEBPA*^BI^ subgroup, two of them died of progressive disease during induction (47). *WT1* mutations are independent poor prognostic factors with a 5-year OS of 35% and EFS of 22% in children (48). No *KIT* mutations were identified in the *CEBPA*^POS^ patients.

In the present research, a low OS of the cohort was noted compared with reports of other parts of the world. It is well recognized that in developing countries, survival rates for this disease are low (∼40%). Several other biological and non-biological factors not evaluated in the present study could be contributing to this poor outcome. For instance, a late presentation, malnutrition, high treatment-related mortality, low SES, and high treatment abandonment rates have been recognized (49). The SES analysis performed, based on the mother years of study as an SES indicator, no statistically significant association was detected between *CEBPA*^POS^ positive cases and a low SES (*p* = 0.99) or between a low SES and a poor outcome (death) (*p* = 0.76; Results of the analysis are shown in Supplementary Table 1). SES does not seem to be a confounding factor affecting the results of this study.

This study has several limitations that must be considered to interpret the findings. Cytogenetic/FISH characterization is not routinely performed in most Mexican public institutions; it was not performed in these patients impairing the risk stratification and precluding further analysis aimed to evaluate the impact of *CEBPA* mutations on the normal karyotype subgroup. This study is a retrospective analysis of a heterogeneous group of patients treated according to four different protocols in eight different institutions. Although this study is the most extensive series of Mexican AML patients analyzed so far, the number of patients is small. Thus, it would reduce the statistical power to detect additional differences in the distribution of clinical features between positive and negative *CEBPA* patients.

However, these limitations do not seem to explain the significant reduction in OS in the *CEBPA*^POS^. The lack of cytogenetic information at diagnosis and the use of different treatment regimens are common to the whole cohort of patients, not affecting the *CEBPA*^POS^ patients exclusively. The mutational status of *CEBPA* gene was unknown to the physician at the moment of choosing treatment protocols and during the whole course of the disease; therefore, a differential bias in clinical management affecting only the *CEBPA*^POS^ group is unlikely. *CEBPA*^POS^ patients were treated in 4 different hospitals. Therefore, a possible “hospital-effect” on OS of *CEBPA*^POS^ patients is also unlikely.

## Conclusion

This study identifies the mutational landscape of the *CEBPA* gene in Mexican pediatric *de novo* AML patients and is the first to evaluate the impact on OS. The results suggest an adverse effect of *CEBPA* mutations in OS, compared with the good prognosis associated with *CEBPA* mutations in adults. Several molecular analyses support that pediatric and adult AML are different clinical and biological entities (50); therefore, the biomarkers identified in adults must be separately validated in the pediatric population before they can be confidently used for clinical management of the disease. Further prospective analyses of extensive series of well-characterized patients are needed to define the clinical impact of the *CEBPA* mutational status in pediatric *de novo* AML.

## Nomenclature

*Resource Identification Initiative*

Cite this (ClinVar, RRID:SCR_006169)

URL: http://www.ncbi.nlm.nih.gov/clinvar/

Cite this**(Genome Aggregation Database, RRID:SCR_01 4964)**

URL: http://gnomad.broadinstitute.org/

Cite this **(COSMIC - Catalogue of Somatic Mutations in Cancer, RRID:SCR_002260)**

URL:http://cancer.sanger.ac.uk/cancergenome/projects/cosmic/

Cite this **(dbSNP, RRID:SCR_002338)**

URL: http://www.ncbi.nlm.nih.gov/SNP/

## Data Availability Statement

The datasets presented in this study can be found in online repositories. The names of the repository/repositories and accession number(s) can be found in the article/Supplementary Material.

## Ethics Statement

The studies involving human participants were reviewed and approved by Ethics Committee of the National Institute of Genomic Medicine, Mexico City, Mexico, (document number 28-2015-1). Written informed consent to participate in this study was provided by the participants or their legal guardian/next of kin.

## Author Contributions

CM obtained the results, analyzed data, and drafted the manuscript. KC, LLF, MJ, AMu, and BV obtained and validated the results. HF validated the results and analyzed statistical data. JN, EJ, VB, JT, JF, JMa, MM, AMe, LE, JP, RE, LF, RA, MP, OS, HR, SJ, PG, and JMe collected clinical data and cared for patients. CA carried out the general supervision, the acquisition of funds, designed the study, obtained and validated the results, analyzed the data, and drafted the manuscript. All authors contributed to the article and approved the submitted version.

## Conflict of Interest

The authors declare that the research was conducted in the absence of any commercial or financial relationships that could be construed as a potential conflict of interest.

## Publisher’s Note

All claims expressed in this article are solely those of the authors and do not necessarily represent those of their affiliated organizations, or those of the publisher, the editors and the reviewers. Any product that may be evaluated in this article, or claim that may be made by its manufacturer, is not guaranteed or endorsed by the publisher.

## Footnotes

1
https://www.ncbi.nlm.nih.gov/clinvar/

2
http://gnomad.broadinstitute.org

3
https://cancer.sanger.ac.uk/cosmic

4
https://www.ncbi.nlm.nih.gov/snp/

5
https://cancer.sanger.ac.uk/cosmic/gene/analysis?ln=CEBPA#variants
