# Supplementary material for: Mutational Landscape of CEBPA in Mexican Pediatric Acute Myeloid Leukemia Patients: Prognostic Implications
Source: Front Pediatr. 2022 Jul 11;10:899742. doi: 10.3389/fped.2022.899742 (PMC9367218; doi:10.3389/fped.2022.899742)
Supplement: Supplementary file 1 [file Data_Sheet_1.pdf]

Supplementary material

1.0 Supplementary tables

To analyze the socioeconomic status (SES) by maternal years of education as an indicator (Table S1), the categorization used was: (0–9 years (low SES), 9.1–12.9 years [reference category], ≥13 years of education (high SES)).

Two-sided Fisher's exact test was used, and a *p*-value < 0.05 was considered statistically significant. Calculations were performed with the SPSS software package, SPSS v21 (Chicago, IL, USA).

Table S1. Analysis of the socioeconomic status in the study population. In (A) was compared the 3 SES levels with *CEBPA*^{POS} and *CEBPA*^{NEG} patients, and the results of association were not statistically significant (*p*=0.99). In (B) were compared the 3 SES levels with the death variable (yes or not), and the results of association were not statistically significant (*p*=0.76).

(A)

			CEBPA status		Total
			negative	positive	
Socioeconomic status by maternal education	9.1 - 12.9 years of study	Recuento	18	2	20
		% dentro de CEBPA status	25.0%	28.6%	25.3%
	0 - 9 years of study	Recuento	35	3	38
		% dentro de CEBPA status	48.6%	42.9%	48.1%
	more than 13 years of study	Recuento	19	2	21
		% dentro de CEBPA status	26.4%	28.6%	26.6%
Total		Recuento	72	7	79
		% dentro de CEBPA status	100.0%	100.0%	100.0%

Fisher´s exact test: 0.99

(B)

			Death		Total
			no	yes	
Socioeconomic status by maternal education	9.1 - 12.9 years of study	Recuento	13	7	20
		% dentro de Death	28.3%	21.2%	25.3%
	0 - 9 years of study	Recuento	22	16	38
		% dentro de Death	47.8%	48.5%	48.1%
	more than 13 years of study	Recuento	11	10	21
		% dentro de Death	23.9%	30.3%	26.6%
Total		Recuento	46	33	79
		% dentro de Death	100.0%	100.0%	100.0%

Fisher´s exact test: 0.76

Chi-square test: 0.71

Table S2. Novel mutations of the CEBPA gene and their classification according to the ACMG criteria, in Mexican patients with pediatric AML.

Patient ID	Coding consequence	cDNA (NM_004364)	Protein consequence	VF(%)	COSMIC ID	ACMG Varsome
M160	inframe_24	c.918_919ins24	p.Arg306_Asn307ins8	42.8	novel	Likely Pathogenic (PM2, PM4, PM1, PP3)
M138	inframe_3	c.946_947insGGA	p.Glu316delinsGlyLys	48.2	novel	Pathogenic (PM1, PM2, PM4, PP3)
M173	frameshift	c.180_183delGTCC	p.Ile62Thrfs*97	44.8	novel	Pathogenic (PVS1, PM2)
M173	inframe_6	c.926_932delAGACGCAinsT	p.Glu309_Gln311delinsVal	42.7	novel	Pathogenic (PM1, PM2, PM4, PP3)
M162	frameshift	c.292delA	p.Thr98Argfs*62	94.4	novel	Likely Pathogenic (PVS1, PM2)
M132	frameshift	c.426delG	p.Arg142Serfs*18	48.7	novel	Likely Pathogenic (PVS1, PM2)
M168	inframe_3	c.334_336delCCC	p.Pro112del	1.3	novel	VUS (PM2, PM4)
M183	inframe_3	c.564_566dupGCC	p.Pro189dup	1.2	novel	VUS (PM2, PP3)

Abbreviations: VF: Variant Fraction. COSMIC: Catalogue Of Somatic Mutations In Cancer. ACMG: American College of Medical Genetics and Genomics. NM_004364 was used for variant annotation

2.0 Supplementary figures

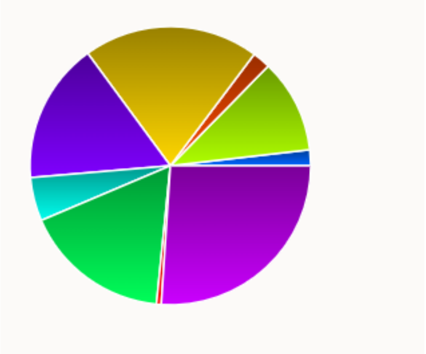

Colour	Mutation type	Number of samples (%)
	Nonsense substitution	37 (2.27%)
	Missense substitution	219 (13.45%)
	Synonymous substitution	42 (2.58%)
	Inframe insertion	407 (25.00%)
	Frameshift insertion	329 (20.21%)
	Inframe deletion	102 (6.27%)
	Frameshift deletion	342 (21.01%)
	Complex mutation	11 (0.68%)
	Other	524 (32.19%)
	Total unique samples	1628

Figure S1. Summary of somatic mutations identified in the *CEBPA* gene across different cancers according to the COSMIC database (as of June 2022). Frameshift deletions and inframe insertions, are the most frequent mutations type, representing 46% of all kinds of mutations in CEBPA across different tumors. COSMIC (Catalogue Of Somatic Mutations In Cancer), (https://cancer.sanger.ac.uk/cosmic/gene/analysis?ln=CEBPA#variants).
